# Baseline and Longitudinal MRI Markers Associated With 16-Year Mortality in Patients With Cerebral Small Vessel Disease

**DOI:** 10.1212/WNL.0000000000209701

**Published:** 2024-08-21

**Authors:** Fang Yi, Mina A. Jacob, Jamie I. Verhoeven, Mengfei Cai, Marco Duering, Anil Man Tuladhar, Frank-Erik De Leeuw

**Affiliations:** From the Department of Geriatrics (F.Y.), Xiangya Hospital, Central South University, China; Department of Neurology (M.A.J., J.I.V., A.M.T., F.-E.D.L.), Research Institute for Medical Innovation, Donders Institute for Brain, Cognition and Behaviour, Radboud University Medical Center, the Netherlands; Department of Neurology (M.C.), Guangdong Neuroscience Institute, Guangdong Provincial People's Hospital, Guangdong Academy of Medical Sciences, Southern Medical University, China; and Department of Biomedical Engineering (M.D.), Medical Image Analysis Center (MIAC AG) and qbig, University of Basel, Switzerland

## Abstract

**Background and Objectives:**

Information on whether small vessel disease (SVD) reduces life expectancy is limited. Moreover, the excess mortality risk attributed specifically to SVD compared with controls from the general population has not been evaluated. This study aimed to investigate the baseline and progression of MRI markers of SVD associated with mortality in a 16-year follow-up cohort study and to determine the excess long-term mortality risk of patients with SVD.

**Methods:**

Participants with SVD from the Radboud University Nijmegen Diffusion Tensor and Magnetic Resonance Imaging Cohort (RUN DMC) study (with MRI assessments in 2006, 2011, 2015, and 2020) were followed until their death or December 1, 2021. Adjusted Cox regression analyses and linear mixed-effect regression models were used to investigate the association between MRI markers of SVD and mortality. The excess mortality risk of SVD was calculated by comparing mortality data of the RUN DMC study with the general population matched by sex, age, and calendar year.

**Results:**

200 of 503 (39.9%) participants died during a follow-up period of 15.9 years. Cause of death was available for 182 (91%) participants. Baseline white matter hyperintensity volume (HR 1.3 per 1-SD increase [95% CI 1.1–1.5], *p* = 0.010), presence of lacunes (1.5 [95% CI 1.1–2.0], *p* = 0.008), mean diffusivity (HR 1.1 per 1-SD increase [95% CI 1.1–1.2], *p* = 0.001), and total brain volume (HR 1.5 per 1-SD decrease [95% CI 1.3–1.9], *p* < 0.001) were associated with all-cause mortality after adjusting for age, sex, and vascular risk factors. Total brain volume decrease over time was associated with all-cause mortality after adjusting for age, sex, and vascular risk factors (HR 1.3 per 1-SD decrease [95% CI 1.1–1.7], *p* = 0.035), and gray matter volume decrease remained significant after additionally adjusting for its baseline volume (1.3 per 1-SD decrease [1.1–1.6], *p* = 0.019). Participants with a Fazekas score of 3, presence of lacunes, or lower microstructural integrity had an excess long-term mortality risk (21.8, 15.7, 10.1 per 1,000 person-years, respectively) compared with the general population.

**Discussion:**

Excess long-term mortality risk only exists in patients with severe SVD (Fazekas score of 3, presence of lacunes, or lower microstructural integrity). This could help in assisting clinicians to predict the clinical outcomes of patients with SVD by severity.

## Introduction

Cerebral small vessel disease (SVD) is a diffuse vascular brain disease, characterized by MRI markers including white matter hyperintensities (WMHs), lacunes of presumed vascular origin, and cerebral microbleeds.^1^ SVD has become an increasingly important public health concern because it is related to cognitive decline and dementia, motor impairment, and stroke, eventually leading to loss of functional independency and institutionalization.^2-6^ However, information on whether SVD also reduces life-expectancy is limited. A systematic review showed that baseline conventional MRI markers of SVD (i.e., WMHs, lacunes, and cerebral microbleeds) increased the risk of all-cause mortality, but the findings were not always consistent because of differences in study population, methods of assessing SVD burden on MRI, and length and frequency of follow-ups.^3^

Given that WMH progression is a nonlinear process and highly variable among individuals,^7,8^ a cross-sectional snapshot of the SVD burden might not provide a reliable association with long-term mortality. Knowledge on the dynamic evolution of MRI markers of SVD, including progression of the more sensitive MRI marker of SVD such as diffusion tensor imaging (DTI) markers, may provide more detailed insights into the relation between SVD and mortality.^9,10^ Only few studies so far investigated the relation between SVD progression/changes over time on MRI and mortality, showing that progression of MRI markers of SVD was associated with mortality.^11,12^ However, these studies involved only a single MRI follow-up assessment, which requires additional validation by studies with multiple MRI follow-ups.

In addition, the mortality risk associated with SVD has primarily been evaluated in a dose-response approach, mostly in cohorts of patients with SVD, but these studies did not particularly investigate whether patients with SVD have an excess mortality risk compared with the general population.

We, therefore, aimed to (1) investigate the relation between baseline severity and progression of MRI and DTI markers of SVD and long-term mortality risk during a follow-up of 16 years and (2) to determine the excess mortality risk of patients with SVD compared with age and sex-matched individuals from the general population.

## Methods

### Study Population

This study is part of the Radboud University Nijmegen Diffusion Tensor and Magnetic Resonance Imaging Cohort (RUN DMC) long-term cohort study on individuals with sporadic SVD, which investigates the risk factors and clinical outcomes of SVD. The study rationale and protocol has been reported previously.^13^ In short, we recruited consecutive community-dwelling participants with SVD aged 50–85 years, who were referred to the Radboud University Medical Center Neurology outpatient clinic between October 2002 and November 2006.

**Figure 1:**
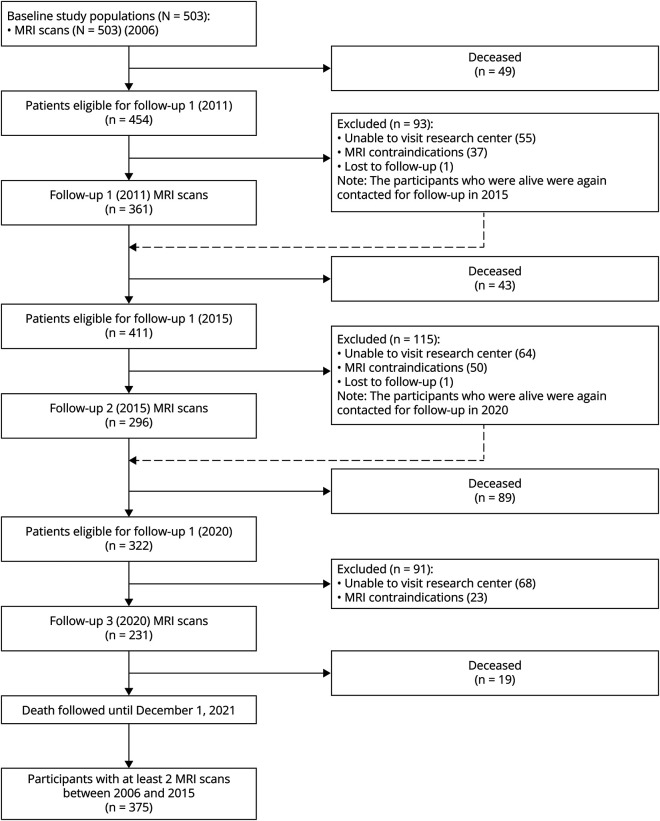
Flowchart of the RUN DMC Study Population Over Time Participants with at least 2 MRI scans between 2006 and 2015, N = 375.

Inclusion criteria were (1) age between 50 and 85 years and (2) SVD on MRI neuroimaging [WMHs and/or lacunar infarct(s)]. As the onset of SVD is often insidious, clinically heterogeneous, and typically with mild symptoms, it has been suggested that the selection of subjects with SVD in clinical studies should be based on these more consistent brain imaging features.^14^ Subsequently, the acute (e.g., transient ischaemic attacks or lacunar syndromes) or subacute clinical symptoms (cognitive, motor (gait) and/or mood disturbances^15^) of SVD were assessed by standardized structured assessments. Patients who were eligible because of a lacunar syndrome were included only >6 months after the event to avoid acute effects on the outcomes. Main exclusion criteria at baseline included presence of dementia, parkinsonism, non–SVD-related white matter lesions (e.g., multiple sclerosis), or a life expectancy of less than 6 months. Follow-up assessments took place in 2011, 2015, and 2020. At all time points, participants completed structured medical questionnaires and underwent physical examinations, motor tests, standardized neuropsychological examinations, and brain MRI. All participants were involved in the association between baseline MRI markers and mortality while those who underwent at least 2 MRI scans between in the follow-up assessments were included in the association between SVD progression and mortality. A flowchart of patient participation in all follow-ups is shown in Figure 1.

### Standard Protocol Approvals, Registrations, and Patient Consents

The Medical Review Ethics Committee region Arnhem‐Nijmegen approved the study, and all participants gave written informed consent.

### Clinical Characteristics

Hypertension was defined as systolic blood pressure (SBP) ≥140 mm Hg and/or diastolic blood pressure (DBP) ≥90 mm Hg and/or the use of blood pressure–lowering agents. SBP/DBP was measured 3 times (separated by 1–2 minutes) in the supine position after 5 minutes of rest during each visit. Diabetes mellitus and hypercholesterolemia were present if the participant was taking oral glucose-lowering drugs or insulin or lipid-lowering drugs, respectively. Information on smoking status was dichotomized into ever (current or former) or never smoking. Structured questionnaires were used to identify atrial fibrillation and cardiovascular and malignant diseases in the medical history. A history of cardiovascular diseases was defined as the presence of an ischemic stroke, hemorrhagic stroke, myocardial infarction, coronary artery bypass grafting, percutaneous coronary intervention, and/or peripheral arterial disease. A history of malignancy was defined as the presence of any malignant neoplasm. This information was subsequently retrieved by the patient's treating physician or from the medical records and was verified thereafter.

### MRI Protocol

Images were acquired on 1.5-Tesla MRI (2006: Siemens, Magnetom Sonata; 2011 and 2015: Siemens, Magnetom Avanto) and included the following whole-brain scans: 3D T1-weighted magnetization prepared rapid acquisition gradient echo(es) (MPRAGE) (isotropic voxel size 1.0 mm^3^); transversal fluid-attenuated inversion recovery (FLAIR) (2006: in-plane resolution 0.5 × 0.5 mm, slice thickness 5.0 mm, interslice gap 1.0 mm; 2011 and 2015: in-plane resolution 0.5 × 0.5 mm, slice thickness 2.5 mm, interslice gap 0.5 mm); transversal T2*-weighted gradient echo sequence (2006: in-plane resolution 1.3 × 1.0 mm, slice thickness 5.0 mm, interslice gap 1.0 mm), and diffusion MRI (2006: isotropic voxel size 2.5 mm, 4 unweighted scans, 30 diffusion-weighted images at b-value 900 s/mm^2^; 2011 and 2015: isotropic voxel size 2.5 mm, 7 unweighted scans, 61 diffusion-weighted images at b-value 900 s/mm^2^). The same 8-channel head coil was used all 3 time points. Full acquisition details have been described previously.^13^ Because there is an MRI upgrade in 2020 (3.0-Tesla), we decided to omit MRI data of follow-up in 2020 to mitigate the long-term bias.

### Brain Volumetry

Gray matter volume (GMV), white matter volume (WMV), and CSF volume were assessed using Statistical Parametric Mapping, SPM12^16^ unified segmentation routines on the T1-weighted MPRAGE images corrected for WMH, as described in detail previously.^10^ Total brain volume (TBV) was calculated by summing GMV and WMV, and intracranial volume (ICV) was calculated further by summing CSF volumes. To correct for interscan effects, we corrected for differences in ICV between baseline and follow-up. We normalized all volumes to baseline ICV to account for head size.

### MRI Markers of SVD

The rating of MRI markers of SVD was based on the Standards for Reporting Vascular Changes on Neuroimaging (STRIVE-2) criteria.^1^ WMH volumes were calculated by a semiautomatic WMH segmentation method, described in detail elsewhere.^17^ In short, after intensity standardization, a computer-aided detection system was used to better detect WMHs of all size based on a two-stage learning approach. Segmentations were visually checked for segmentation errors by 1 trained rater, blinded for clinical data. To minimize the effects of changes in FLAIR acquisition sequence parameters, follow-up FLAIR images were resliced to match baseline slice thickness using FMRIB's Linear Image Registration Tool, part of FSL.^18^ WMH volumes were normalized to ICV. In addition, for clinical generalizability, severity of WMH was also classified according to the modified Fazekas scale.^19^ Lacunes and cerebral microbleeds were rated manually on FLAIR/T1-weighted and T2*-weighted MRI scans, respectively. Incident lacunes were identified by difference maps.^20^ In short, all follow-up images were registered to the baseline scans; difference images were generated by subtracting the registered and intensity-normalized baseline T1 and FLAIR images from the corresponding T1 and FLAIR images at the follow-ups. These MRI markers were rated by 2 trained raters blinded for clinical data with excellent inter-rater agreement in a random sample of 10% of the scans (weighted kappa of 0.87 and 0.95, respectively, for the number of lacunes and 0.85 and 0.86 for the number of cerebral microbleeds).^21^

### Diffusion Tensor Imaging Processing

For all time points (2006, 2011 and 2015), all diffusion-weighted images were denoised using a local principal component analysis filter^22^ and corrected for cardiac, head motion, and eddy current artifacts simultaneously using the “PATCH” algorithm as described previously.^23^ Using the “dtifit” in FSL, we created fractional anisotropy and mean diffusivity (MD) mappings, which were further used to extract mean MD in WM area.^24^

### Mortality

Participants were followed until their death or December 1, 2021. Information on vital status was retrieved from the Dutch Municipal Personal Records database. All-cause mortality was the primary outcome of this study. Information on the cause of mortality was obtained from general practitioners or treating physicians at the time of death. According to the International Statistical Classification of Diseases and Related Health Problems, *Tenth Revision (ICD-10)*, we classified cause of death based on the underlying cause of death, defined as the condition which initiated the sequence of morbid events leading to death. The underlying condition is usually a chronic disease leading to fatal complications. Causes of death were categorized as follows: ischemic stroke, intracerebral hemorrhage, cardiac cause, other vascular event, dementia, malignancy, infections, and other determined cause. The “other vascular event” group included deaths that were presumably vascular but did not meet the criteria for fatal stroke or cardiac cause. Stroke-related death, as analyzed in this study, included ischemic stroke and intracerebral hemorrhage.

Mortality data on the general population were extracted from the Dutch Population Registry from January 1, 2006, to December 1, 2021. Details of these registries and linkage procedures have been previously described.^25^

### Statistical Analysis

Differences in baseline characteristics between survivors and participants who deceased during follow-up were investigated by using χ^2^ test, Student t-test, or Wilcoxon Mann-Whitney *U* test, where appropriate.

Cumulative all-cause mortality was estimated by using Kaplan-Meier analysis and stratified for severity of the MRI markers of SVD. Differences between the lowest and highest quartiles (i.e., WMH volumes, MD of white matter, GMV, and WMV) and the presence vs absence (i.e., lacunes and microbleeds) were tested by using log-rank test.

Cox regression analyses were used to investigate the association between baseline MRI markers of SVD and mortality, after adjustment for age, sex, vascular risk factors (i.e., hypertension, smoking, diabetes mellitus, hypercholesterolemia, atrial fibrillation, and history of cardiovascular disease). Similar analyses were also performed for stroke-related, dementia-related, cardiac-related, and other vascular-related mortality; causes of death other than the studied group cause of mortality were considered a competing risk, by using the proportional hazards model of Fine and Gray.^26^ Schoenfeld residuals were investigated to verify the proportionality of hazards. There were no indications that the proportional hazards assumption was violated.

To investigate the association between SVD progression and mortality, we constructed linear mixed-effect models to estimate annualized rate changes of MRI markers, that is, WMH volumes, GMV, WMV, and TBV. The intercept and slope of each participant's linear trajectory were allowed to vary with both fixed and random effects. The fixed effect of time represents the average annualized rate of change of MRI markers across the whole cohort, whereas random effects of intercept and slope per participant can allow for interindividual variability. Slopes for each participant were extracted and used for further analyses. Three Cox regression models were performed: model 1 was adjusted for age and sex; model 2 was adjusted for age, sex, and vascular risk factors (i.e., hypertension, smoking, diabetes mellitus, hypercholesterolemia, atrial fibrillation and history of cardiovascular disease); and model 3 was additionally adjusted for the baseline measure of each MRI marker. Because there was a scanner upgrade of DTI in 2011, changes in MD of the white matter were only obtained between 2011 and 2015. Therefore, we examined the relation between MD progression between 2011 and 2015 and mortality risk after 2015.

To calculate the excess mortality risk of patients with SVD compared with the Dutch general population, we compared mortality data of the RUN DMC study (participants with SVD) with the general Dutch Mortality Database matched by sex, age, and calendar year.^25^ The annual risk of observed mortality within the RUN DMC cohort, as well as the expected mortality in the general population, was calculated using the formula: 1−([1−Ic]^[1/n]^), where n is the number of years after the index event and Ic is the cumulative mortality at n years, obtained by Kaplan-Meier analysis.^27^

Standardized mortality ratios (SMRs) were calculated by dividing the observed deaths in the cohort by the expected deaths of their peers from the general population for each MRI measure subtype, that is, severity of WMH (Fazekas score 1–3) or MD (high or low), presence of lacunes, or presence of microbleeds. High MD subtype was defined as higher than or equal to the median MD of the participants with SVD. The expected matched mortality rates were retrieved from the Dutch Population Registry Database.^28,29^ The 95% CIs were calculated by assuming a Poisson distribution. In addition, for each subgroup, we calculated an absolute excess number of deaths by taking the difference between the observed and expected deaths, divided by the person-years at risk.

Two-tailed *p* values <0.05 were considered statistically significant. All statistical analyses were performed in R (version 4.1.2).

### Data Availability

The data that support the findings of this study may be made available on reasonable request.

## Results

### Baseline Characteristics

The RUN DMC consisted of 503 participants at baseline (2006) with a mean age of 65.7 (SD 8.8) years. Two participants were censored because they were lost to follow-up because of emigration after 13.2 and 8.8 years. In total, 200 (39.9%) participants died during a follow-up period of 15.9 years (between January 17, 2006, and December 1, 2021). Cause of death was retrieved in 182 (91%) of the 200 deceased participants. Malignancy was the most common cause of death affecting 40 (20%) patients. Regarding vascular-related mortality, 33 (16.5%) were cardiac-related, 20 (10%) were stroke-related, and 12 (6%) were other vascular-related. 32 (16%) participants died of dementia (Table 1). Participants who deceased were older, had higher presence of vascular risk factors (e.g., smoking, atrial fibrillation, hypertension, diabetes), cardiovascular events, and higher burden of SVD on MRI at baseline than the survivors. Differences in baseline characteristics of the study population are presented in Table 2.

**Table 1:** Causes of Mortality in the Whole Study

Cause of death	Number of participants, no. (%) (total n = 200)
Malignancy	40 (20)
Cardiac cause	33 (16.5)
Dementia	32 (16)
Stroke	20 (10)
Ischemic stroke	14 (7)
Intracerebral hemorrhage	6 (3)
Infections	20 (10)
Other determined	19 (9.5)
Unknown	18 (9)
Other vascular	12 (6)
Traumatic injury	6 (3)

**Table 2:** Differences in Baseline Characteristics Between Survivors and Deceased Participants

	Alive (n = 301)	Deceased (n = 200)	*p* Value
Demographic			
Age, y (SD)	61.97 (7.4)	71.32 (7.6)	<0.001^a^
Sex, female (%)	143 (47.5)	75 (37.5)	0.034^b^
Vascular risk factors			
BMI, kg/m^2^ (SD)	27.12 (3.97)	27.23 (4.33)	0.787^a^
Smoking, N (%)	195 (64.8)	157 (78.5)	0.001^b^
Atrial fibrillation, N (%)	14 (4.7)	26 (13.0)	0.001^b^
Hypertension, N (%)	200 (66.4)	167 (83.5)	<0.001^b^
Diabetes, N (%)	29 (9.6)	45 (22.5)	<0.001^b^
Hypercholesterolemia, N (%)	126 (41.9)	111 (55.5)	0.004^b^
Comorbidity			
Cardiovascular events, N (%)	126 (41.9)	121 (60.5)	<0.001^b^
Malignancies, N (%)	27 (9.0)	26 (13.0)	0.198^b^
MRI characteristics			
WMH, ml [IQR]^d^	2.26 [0.79, 6.93]	8.34 [3.03, 17.35]	<0.001^c^
Presence of lacunes, N (%)	54 (17.9)	83 (41.5)	<0.001^b^
Presence of cerebral microbleeds, N (%)^e^	41 (13.8)	42 (21.1)	0.042^b^
MD white matter 10^−3^ mm^2^/s^f^	0.87 (0.04)	0.93 (0.15)	<0.001^a^
TBV, mL (SD)^d^	1,093.62 (66.37)	1,011.99 (73.92)	<0.001^a^
GMV, mL (SD)^d^	624.80 (45.71)	577.82 (49.66)	<0.001^a^
WMV, mL (SD)^d^	469.05 (37.63)	433.00 (49.02)	<0.001^a^

Abbreviations: BMI = body mass index; GMV = gray matter volume; IQR = interquartile range; MD = mean diffusivity; TBV = total brain volume; WMH = white matter hyperintensity; WMV = white matter volume.

Data represent numbers (%), means ± SD, or medians (IQR). In total, 2 participants were lost to follow-up and excluded from baseline analyses.

a Student *t* test.

b χ^2^ test.

c Mann-Whitney *U* test.

d Brain volumes are represented as normalized to the total intracranial volume.

e Four participants excluded because of insufficient scan quality.

f Three participants excluded because of insufficient scan quality.

### Association Between Baseline SVD Markers and Mortality

**Figure 2:**
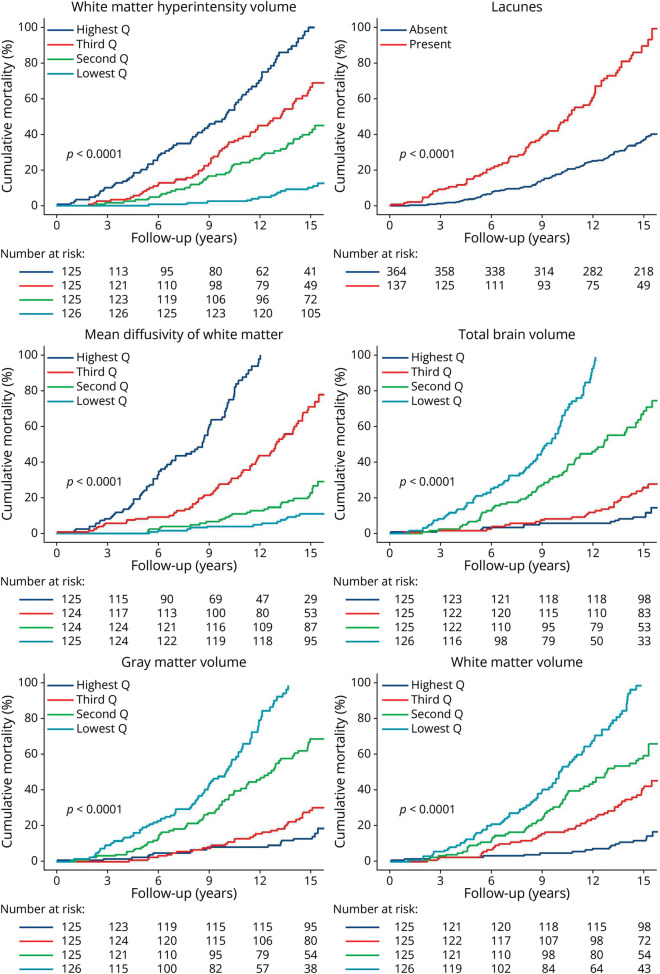
Cumulative Mortality Stratified by Baseline MRI Markers Cumulative mortality was estimated using Kaplan-Meier analysis; this was stratified for different MRI markers at baseline. The differences between the lowest and highest quartiles and presence vs absence were estimated with the log-rank test. Q indicates quartile.

All-cause 16-year mortality probability was highest in participants with the presence of lacunes; the highest WMH volume or MD; and the lowest TBV, GMV, and WMV, at baseline (Figure 2). Baseline MRI markers of SVD were associated with all-cause mortality after adjusting for age, sex, and vascular risk factors (reported as HR [95% CI]): WMH (1.3 per 1-SD increase [1.1–1.5], *p* = 0.010), presence of lacunes (1.5 [1.1–2.0], *p* = 0.008), MD (1.1 per 1-SD increase [1.1–1.2], *p* = 0.001), and TBV (1.5 per 1-SD decrease [1.3–1.9], *p* < 0.001; Table 3).

**Table 3:** Association Between Baseline MRI Markers and All-Cause Mortality

	Model 1		Model 2	
MRI measures	HR (95% CI)	*p* Value	HR (95% CI)	*p* Value
WMH (mL), per 1-SD increase^a^	1.35 (1.14–1.61)	0.001	1.26 (1.06–1.51)	0.010
Presence of lacunes	1.75 (1.31–2.34)	<0.001	1.50 (1.11–2.03)	0.008
Presence of cerebral microbleeds	1.07 (0.76–1.51)	0.712	1.06 (0.75–1.50)	0.752
MD white matter, 10^−3^ mm^2^/s, per 1-SD increase	1.14 (1.06–1.22)	<0.001	1.13 (1.05–1.22)	0.001
TBV (mL), per 1-SD decrease^a^	1.64 (1.35–2.00)	<0.001	1.54 (1.27–1.85)	<0.001
GMV (mL), per 1-SD decrease^a^	1.37 (1.14–1.67)	0.001	1.30 (1.08–1.56)	0.008
WMV (mL), per 1-SD decrease^a^	1.33 (1.16–1.54)	<0.001	1.28 (1.10–1.49)	0.001

Abbreviations: GMV = gray matter volume; HR = hazard ratio; MD = mean diffusivity; TBV = total brain volume; WMH = white matter hyperintensity; WMV = white matter volume.

Model 1: Adjusted for age and sex.

Model 2: Adjusted for age, sex, and vascular risk factors (smoking, hypertension, diabetes mellitus, hypercholesterolemia, atrial fibrillation, and history of cardiovascular disease).

a Brain volumes are represented as normalized to total intracranial volume.

Higher WMH (1.9 per 1-SD increase [1.1–3.6], *p* = 0.044) and MD (1.3 per 1-SD increase [1.2–1.3], *p* < 0.001) and lower TBV (2.4 per 1-SD decrease [1.4–4.1], p < 0.001) and WMV (1.7 per 1-SD decrease [1,2–2.4], *p* = 0.002) were specifically associated with stroke-related mortality after adjustments for age and sex, but significance was lost for WMH after additionally adjusting for vascular risk factors. However, presence of lacunes (2.4 [1.1–5.3], *p* = 0.039) and lower TBV (1.7 per 1-SD decrease [1.1–2.8], p = 0.033) and WMV (1.6 per 1-SD decrease [1.2–2.1], *p* = 0.001) were associated with dementia-related mortality, as presented in eTable 1. None of the studied MRI markers of SVD were associated with non-stroke vascular-related death (data not shown).

### Association Between SVD Progression and Mortality

A total of 375 participants underwent at least 2 MRI scans during any of the follow-ups in 2006, 2011, and 2015. Of those, 106 participants died, of whom 11 (10.4%) were stroke-related deaths and 19 (17.9%) were dementia-related deaths (eTable 2). Differences in baseline characteristics between the participants who dropped out and those who remained in the study are presented in eTable 3. In the multivariable Cox regression models (Table 4), progression of MRI markers of SVD over time did not show an association with all-cause mortality. TBV decrease (1.3 per 1-SD decrease [1.1–1.7], *p* = 0.035) and GMV decrease (1.2 per 1-SD decrease [1.1–1.5], *p* = 0.038) were significantly associated with all-cause mortality after adjusting for age, sex, and vascular risk factors, and only GMV decrease (1.3 per 1-SD decrease [1.1–1.6], *p* = 0.019) remained significant after additionally adjusting for their baseline volume. There was no relation between progression of the studied MRI markers and stroke-related mortality (eTable 4). Incident lacunes (5.4 [1.7–16.9], *p* = 0.003), TBV decrease (3.7 per 1-SD decrease [1.8–7.5], *p* < 0.001), and GMV decrease (2.0 per 1-SD decrease [1.3–3.1], *p* = 0.001) were associated with dementia-related death after adjustment of age, sex, vascular risk factors, whereas significance was lost for WMV decrease after additionally adjusting for their baseline volume (eTable 5).

**Table 4:** Association Between SVD Progression and All-Cause Mortality

	Model 1		Model 2		Model 3	
MRI measures	HR (95% CI)	*p* Value	HR (95% CI)	*p* Value	HR (95% CI)	*p* Value
WMH increase (mL/y) per 1-SD increase^a^	1.07 (0.86–1.33)	0.565	0.97 (0.77–1.22)	0.796	0.72 (0.47–1.10)	0.128
Incident lacunes	1.96 (1.10–3.48)	0.022	1.84 (1.02–3.33)	0.044	1.75 (0.97–3.18)	0.065
Incident cerebral microbleeds	1.49 (0.87–2.57)	0.149	1.31 (0.74–2.32)	0.361	1.33 (0.75–2.38)	0.332
MD white matter increase (10^−3^ mm^2^/s/y) per 1-SD increase^b^	1.01 (0.79–1.30)	0.916	0.97 (0.75–1.27)	0.850	0.98 (0.74–1.28)	0.859
TBV decrease (mL/y) per 1-SD decrease^a^	1.36 (1.08–1.72)	0.009	1.29 (1.02–1.66)	0.035	1.26 (0.98–1.61)	0.067
GMV decrease (mL/y) per 1-SD decrease^a^	1.25 (1.02–1.51)	0.031	1.23 (1.01–1.51)	0.038	1.28 (1.04–1.56)	0.019
WMV decrease (mL/y) per 1-SD decrease^a^	1.12 (0.90–1.38)	0.278	1.06 (0.84–1.33)	0.620	1.03 (0.80–1.31)	0.826

Abbreviations: GMV = gray matter volume; HR = hazard ratio; MD = mean diffusivity; TBV = total brain volume; WMH = white matter hyperintensity; WMV = white matter volume.

Model 1: Adjusted for baseline age, sex.

Model 2: Adjusted for baseline age, sex and vascular risk factors (smoking, hypertension, diabetes mellitus, hypercholesterolemia, atrial fibrillation, and history of cardiovascular disease).

Model 3: Additionally adjusted for each baseline MRI measurement.

a Brain volumes are represented as normalized to total intracranial volume.

b Progression of MD white matter between 2011 and 2015 and mortality after 2015.

### Excess Mortality

**Figure 3:**
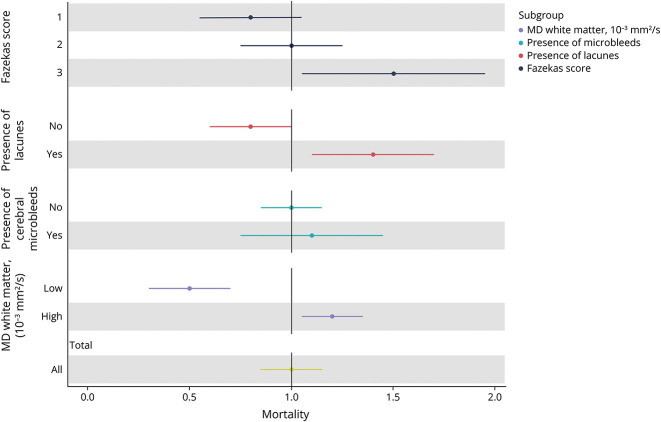
Standardized Mortality Rate in Patients With SVD Compared With the Dutch General Population Standardized mortality ratios were calculated by dividing the observed deaths in the RUN DMC study by the expected deaths of their peers from the Dutch general population (retrieved from the Dutch Population Registry Database) for each MRI measure subtype, that is, severity of WMH (Fazekas score 1–3) or MD (high or low), presence of lacunes or microbleeds. RUN DMC = Radboud University Nijmegen Diffusion Tensor and Magnetic Resonance Imaging Cohort; WMH = white matter hyperintensity.

There was no excess mortality risk for the whole group of participants with SVD compared with their peers from the general population matched by sex, age, and calendar year (SMR: 1.0, 95% CI 0.8–1.1). However, participants with a Fazekas score of 3 had a higher excess mortality risk: SMR: 1.5, 95% CI 1.1–2.0, with an observed mortality rate of 67.2 per 1,000 person-years vs an expected mortality rate of 45.4 per 1,000 person-years and an excess mortality rate of 21.8 per 1,000 person-years. This excess mortality risk was also observed in participants with prevalent lacunes (SMR: 1.4, 95% CI 1.1–1.7) and for participants with higher MD (SMR: 1.2, 95% CI 1.1–1.4). The results are presented in Table 5 and Figure 3.

**Table 5:** Excess Risk of Mortality in Patients With SVD Compared With the General Population

	Patient-years at risk	Observed death	Observed deaths per 1,000 person-years	Expected death	Expected deaths per 1,000 person-years	Excess rate per 1,000 person-years	Standardized mortality rate(95% CI)
Total	5,819.5	168	28.9	169.5	29.1	−0.2	1.0 (0.8–1.1)
Fazekas score							
1	4,071.8	78	19.2	94.2	23.1	−3.9	0.8 (0.6–1.1)
2	1,137.3	49	43.1	47.6	41.9	1.2	1.0 (0.8–1.3)
3	610.5	41	67.2	27.7	45.4	21.8	1.5 (1.1–2.0)
Presence of lacunes							
No	4,409.2	93	21.1	116.6	26.4	−5.3	0.8 (0.6–1.0)
Yes	1,410.3	75	53.2	52.9	37.5	15.7	1.4 (1.1–1.7)
Presence of cerebral microbleeds							
No	4,919.2	130	26.4	135.1	27.5	−1.1	1.0 (0.8–1.1)
Yes	900.3	38	42.2	34.4	38.2	4.0	1.1 (0.8–1.5)
MD white matter, 10^−3^ mm^2^/s^a^							
Low	3,211.6	27	8.4	52.4	16.3	−7.9	0.5 (0.3–0.7)
High	2,567.5	141	54.9	114.9	44.8	10.1	1.2 (1.1–1.4)

a Categorization of the median diffusivity (MD) into low vs high was based on the median.

## Discussion

In this study, we found that baseline MRI markers of SVD significantly increased the 16-year risk of mortality while progression of these SVD markers was not associated with all-cause mortality nor stroke-related mortality. However, TBV and GMV decrease were associated with all-cause mortality. Incident lacunes, as well as decrease in TBV and GMV, were associated with dementia-related mortality over time. There was an excess long-term mortality risk in participants with presence of severe WMH (Fazekas 3), presence of lacunes, and lower microstructural integrity, compared with their peers from the general population.

In addition to the previous studies primarily investigating conventional MRI markers of SVD, we have shown that impaired microstructural integrity (i.e., MD of white matter) is also associated with long-term mortality. Of interest, WMH burden and lower microstructural integrity were associated with stroke-related mortality, but not with non-stroke vascular-related death. This is in line with the 3-City Study which showed that WMH was associated with stroke incidence, but not with other non-stroke vascular incident events.^30^ However, 2 other studies found an association between WMH and vascular death, but this was also mainly driven by stroke-related death.^31,32^ This might suggest that MRI markers of SVD actually represent distinctive and specific cerebrovascular pathophysiologic processes (e.g., neuroinflammation),^33^ rather than serving as indicators of a broad and generalized vascular damage. However, this hypothesis warrants further detailed investigation.

We did not observe a significant association between progression of MRI markers of SVD (e.g., WMH progression or incident lacunes) and all-cause mortality, total vascular mortality and stroke-related mortality, which is to some extent in contrast with the 2 previous studies (PROSPER and the Cardiovascular Health Study) investigating WMH progression over time.^11,12^ Several explanations could be put forward for these contrasting findings. First, the participants in our cohort were much younger at baseline than the other studies (65.7 years vs 74.5 years and 74.1 years, respectively).^11,12^ Given that our annualized WMH progression rate was 0.31 mL/y extracted from a linear mixed effects model, which was even larger than the progression reported in the PROSPER study (0.20 mL/y), this raises the possibility that age and its related selective survival may have altered the relative importance of SVD (progression) in relation to mortality: Patients with the most severe baseline SVD, who have the highest SVD progression over time,^7^ are also at the highest risk of being lost to follow-up because of their higher (vascular) frailty, leaving a residual group of patients with relatively mild SVD. As a result, the lost to follow-up patients with severe SVD could have attenuated the actual effect of SVD progression on mortality. This translated in that nearly 50% of the causes of death were not related to vascular events or dementia. Second, the 2 previous studies had only 1 follow-up assessment and a much shorter mean follow-up interval time between the 2 MRI scans (2.5 and 5 years) than our study (2 follow-up time points with a median of 9-year follow-up). Given the heterogeneous temporal trajectory of SVD, with progression alternated by potential regression over time,^8,34,35^ it is plausible that the longer follow-up period in our study facilitated the observation of SVD regression (5.4%), which could mitigate the effect of total progression on the outcome. Of note, the proportion of subjects with WMH regression varied considerably ranging from 2% to 37% in different populations.^36^ Third, they also did not adjust for baseline WMH severity or lacunes, which may strongly influence the results, because baseline SVD severity is strongly associated with SVD progression.^7,37^ Fourth, the Cardiovascular Health Study included all incident infarcts larger than 3 millimeters, not only lacunes but also territorial infarcts.^12^ Given that infarcts larger than 15 millimeters do not qualify a lacune according to STRIVE-2 criteria and are most likely to be attributed to cardioembolism or large artery disease,^38^ their results may thereby increase the odds of a cardiovascular death.

We found that GMV and TBV decrease over time were associated with all-cause mortality, with GMV decrease showing a stronger effect on death. Although lower baseline brain volume has been demonstrated to be associated with all-cause mortality in patients with SVD,^39^ our study showed that the progression of brain atrophy was also significantly associated with all-cause mortality. This may indicate that overall neurodegeneration, which also captures other causes of neurodegenerative diseases, rather than single MRI markers of SVD, may be important in predicting risk of mortality. Future investigation is still warranted to validate our findings. Progression of brain atrophy and incident lacunes were also related to dementia-related death, which may suggest that SVD is more likely to (first) lead to cognitive impairments and dementia, instead of fatal events.

Progressive impaired microstructural integrity (i.e., MD of white matter) was not related to mortality in this study. Because there was a scanner upgrade of DTI between 2006 and 2011, we thus could only reliably assess MD change between 2011 and 2015. Because SVD progression is a slow process, a 4-year interval may not be sufficient to detect relevant changes in the microstructural integrity in this cohort with participants with mild SVD. Besides, this result should be interpreted with caution because of the relatively small sample size and limited statistical power.

One of the main findings of this study is that although the observed mortality rate in our entire cohort was similar to the expected rate in the general population, an excess long-term mortality risk was observed in patients with the highest severity of SVD (in terms of WMH, impaired microstructural integrity, and presence of lacunes), compared with their age and sex-matched peers. Owing to the generally low burden of SVD in presumed healthy populations,^40^ our results underline that this excess mortality risk is most likely to be attributed to SVD or its related risk factors.

Our results offer valuable insights for clinicians, suggesting that SVD should be interpreted distinctively regarding its severity and its clinical consequences. Specifically, there may be opportunities for health benefit within the subgroup with highest SVD burden, in contrast to patients with mild to moderate SVD who rarely progress and do not exhibit an excess long-term mortality risk compared with their peers from the general population.^7^ This may also be of great importance to aid in reducing the sample size of randomized controlled trials in treatment of patients with SVD, by only including participants with severe SVD, for example, WMH Fazekas score of 3.

Our study has some limitations. First, although we have minimized the risk of measurement errors by reslicing follow-up scans to the same resolution as baseline FLAIR scans, this approach still did not take into account the potential influence of different field strengths, potentially leading to higher estimated rates of WMH progression because of thinner slices and higher field strength at the follow-ups. However, these differences are almost inevitable with improvements in hardware over time during a follow-up design, especially for very long follow-up studies. Second, the association between mortality and progression of SVD MRI markers may have been underestimated because of attrition bias: Those dropped out during follow-up assessments were older, had higher presence of vascular risk factors (i.e., hypertension, diabetes, hypercholesterolemia), and higher burden of SVD on MRI at baseline than those who remained in the study. Third, we did not have available imaging data of the general population, which prevented us from assessing their matched severity of SVD burden when quantifying the excess risk. However, the reported SVD severity seen on MRI in elderly individuals from the general population is generally low.^40-42^ Besides, the COVID-19 pandemic's overlap with our study time line introduces a complex variable, potentially influencing mortality rates and affecting the generalizability of our results. Despite the comprehensive nature of our data set, the lack of granularity regarding the cause of death during the pandemic period represents a notable limitation. This gap in data necessitates a cautious approach in interpreting our findings, especially in relation to mortality rates. Finally, most participants in this study were of White ancestry, thus further research is required to verify whether our findings apply to other ethnic groups as well.

Major strengths of this study include the large sample size, the longitudinal design up to 16 years, and 3 follow-ups including MRI scans. To date, this study is the first to report the role of SVD progression on mortality risk in a prespecified cohort of patients with sporadic SVD comprehensively, including incident microbleeds and diffusion tensor imaging changes. Moreover, we have complete information on causes of death in our cohort (>91% of 200 cases). In addition, all neuroimaging data were analyzed by raters blinded to clinical information and were assessed reliably and sensitively.

In conclusion, patients with severe WMH (Fazekas score of 3), prevalent lacunes, or impaired microstructural integrity had a higher 16-year mortality risk than the age and sex-matched general population. Progression of diffuse cerebral atrophy and not of SVD markers was related to increased mortality in patients with SVD. Further research is needed on delaying the development of SVD and its clinical consequences.

## Appendix Authors

**Table:**

Name	Location	Contribution
Fang Yi, PhD, MD	Department of Geriatrics, Xiangya Hospital, Central South University, China	Drafting/revision of the manuscript for content, including medical writing for content; major role in the acquisition of data; study concept or design; analysis or interpretation of data
Mina A. Jacob, MD	Department of Neurology, Research Institute for Medical Innovation, Donders Institute for Brain, Cognition and Behaviour, Radboud University Medical Center, the Netherlands	Drafting/revision of the manuscript for content, including medical writing for content; major role in the acquisition of data; study concept or design; analysis or interpretation of data
Jamie I. Verhoeven, MD	Department of Neurology, Research Institute for Medical Innovation, Donders Institute for Brain, Cognition and Behaviour, Radboud University Medical Center, the Netherlands	Drafting/revision of the manuscript for content, including medical writing for content; analysis or interpretation of data
Mengfei Cai, MD, PhD	Department of Neurology, Guangdong Neuroscience Institute, Guangdong Provincial People's Hospital, Guangdong Academy of Medical Sciences, Southern Medical University, China	Drafting/revision of the manuscript for content, including medical writing for content; major role in the acquisition of data; analysis or interpretation of data
Marco Duering, MD, PhD	Department of Biomedical Engineering, Medical Image Analysis Center (MIAC AG) and qbig, University of Basel, Switzerland	Drafting/revision of the manuscript for content, including medical writing for content
Anil Man Tuladhar, MD, PhD	Department of Neurology, Research Institute for Medical Innovation, Donders Institute for Brain, Cognition and Behaviour, Radboud University Medical Center, the Netherlands	Drafting/revision of the manuscript for content, including medical writing for content; major role in the acquisition of data; analysis or interpretation of data
Frank-Erik De Leeuw, MD, PhD	Department of Neurology, Research Institute for Medical Innovation, Donders Institute for Brain, Cognition and Behaviour, Radboud University Medical Center, the Netherlands	Drafting/revision of the manuscript for content, including medical writing for content; study concept or design; analysis or interpretation of data

## Glossary

DBP: diastolic blood pressure
DTI: diffusion tensor imaging
FLAIR: fluid-attenuated inversion recovery
GMV: gray matter volume
ICV: intracranial volume
MD: mean diffusivity
MPRAGE: magnetization prepared rapid acquisition gradient echo(es)
RUN DMC: Radboud University Nijmegen Diffusion Tensor and Magnetic Resonance Imaging Cohort
SBP: systolic blood pressure
SMR: standardized mortality ratio
SVD: small vessel disease
TBV: total brain volume
WMH: white matter hyperintensity
WMV: white matter volume
